# Impact of Cushing’s sign in the prehospital setting on predicting the need for immediate neurosurgical intervention in trauma patients: a nationwide retrospective observational study

**DOI:** 10.1186/s13049-016-0341-1

**Published:** 2016-12-09

**Authors:** Tetsuya Yumoto, Toshiharu Mitsuhashi, Yasuaki Yamakawa, Atsuyoshi Iida, Nobuyuki Nosaka, Kohei Tsukahara, Hiromichi Naito, Atsunori Nakao

**Affiliations:** 1 Advanced Emergency and Critical Care Medical Center, Okayama University Hospital, 2-5-1 Kita-ku, Shikata-cho, Okayama-shi, Okayama, 700-8558 Japan; 2 Center for Innovative Clinical Medicine, Okayama University Hospital, 2-5-1 Kita-ku, Shikata-cho, Okayama-shi, Okayama, 700-8558 Japan

**Keywords:** Traumatic brain injury, Hypertension, Bradycardia, Cushing’s sign, Prehospital

## Abstract

**Background:**

Cushing’s reflex usually results from intracranial hypertension. Although Cushing’s sign can implicate severe traumatic brain injury (TBI) in injured patients, no major investigations have been made. The purpose of this study was to assess the predictability of life-threatening brain injury requiring immediate neurosurgical intervention (LT-BI) among trauma patients with Cushing’s sign in the prehospital setting.

**Methods:**

This was a retrospective study using data from the Japan Trauma Data Bank from the period of 2010 to 2014. Patients 16 years old or older with blunt mechanisms of injury who were transported directly from the scene and Glasgow Coma Scale for eye opening of one in the prehospital setting were included. LT-BI was defined as patients requiring burr hole evacuation or craniotomy within 24 h of hospital arrival and patients who were non-survivors due to isolated severe TBI. Prehospital systolic blood pressure (pSBP) and heart rate (pHR) were assessed using area under the receiver operating characteristic curve (AUROC) and multiple logistic regression analysis to predict LT-BI.

**Results:**

Of 6332 eligible patients, 1859 (29%) exhibited LT-BI. AUROC of LT-BI using pSBP and pHR was 0.666 (95% confidence interval (CI); 0.652–0.681, *P* < 0.001), and 0.578 (95% CI; 0.563–0.594, *P* < 0.001), respectively. AUROC of pSBP was the highest among the 60 ≤ pHR ≤ 99 subgroup, of which AUROC was 0.680 (95% CI; 0.662–0.699, *P* < 0.001).

Multiple logistic regression analysis showed that the higher the pSBP and the lower the pHR, the more likely that the patients had LT-BI. In a group with pSBP ≥ 180 mmHg and pHR ≤ 59 beats/min, the odds ratio and 95% CI of LT-BI after adjusting for age, sex, and severity of injuries to other body regions was 4.77 (2.85–7.97), *P* < 0.001 was compared with the reference group, which was defined as patients with normal vital signs.

**Discussion:**

Our study has found that the combination of hypertension and bradycardia, which are the components of Cushing's sign without eye opening in the prehospital setting was a weak but a significant predictor of LT-BI, or death due to possible isolated severe TBI.

**Conclusions:**

Prehospital Cushing’s sign with disturbed level of consciousness in trauma patients was a weak but significant predictor of the need for immediate neurosurgical intervention.

## Background

Cushing’s reflex is a well known phenomenon characterized by hypertension, bradycardia, and respiratory irregularity [1]. It is an alarming sign that may predict the subsequent occurrence of brain herniation and death. Elevated blood pressure in patients with severe traumatic brain injury (TBI) results from intracranial hypertension in order to maintain cerebral perfusion pressure and cerebral blood flow [2]. Systemic hypertension may also be associated with paroxysmal sympathetic hyperactivity [3]. Hypertension in the prehospital setting is predictive of TBI and is associated with higher mortality [4]. Paradoxically, hypotension, which can lead to impaired cerebral perfusion, is also associated with increased mortality in patients with severe TBI [5].

Although hypertension plus bradycardia with severe disturbance of consciousness in the prehospital setting or at admission can implicate severe TBI in trauma patients, there have been no major investigations. The aim of this study was to assess the effectiveness of prehospital Cushing’s sign in predicting life-threatening brain injury requiring immediate neurosurgical intervention (LH-BI) in isolated severe TBI patients, which can be helpful for earlier neurosurgical management and intervention.

## Methods

### Study design and data collection

This was a retrospective nationwide observational study. We analyzed data from the Japan Trauma Data Bank (JTDB), which is similar to trauma databases in Europe and North America [6]. JTDB was established in 2003 with the Japanese Association for the Surgery of Trauma (Trauma Surgery Committee) and the Japanese Association for Acute Medicine (Committee for Clinical Care Evaluation) [7]. The JTDB represents a large national repository of trauma patients. Data are continuously entered into a web-based database using Abbreviated Injury Scale (AIS) 98. As of March 2015, 244 major voluntarily institutions have participated in Japanese trauma care and research. Of 141,060 patients registered from 2009 to 2013, the number of patients of Injury Severity Score (ISS) of 9–15 (39%) category was the most of all categories followed by 16–24 (20%), 1–9 (17%), and 25–40 (14%) [7]. This study was approved by the Okayama University Hospital ethical committee (ID 1607–025).

### Selection of participants and definitions

A total of 139,847 trauma patients were enrolled in the JTDB between January 2010 and December 2014. Patients who were 16 years old or older presenting with a blunt injury transported directly from the scene and Glasgow Coma Scale (GCS) for eye opening of 1 in the prehospital setting were included. Patients who were in cardiac arrest on arrival were excluded. Patients with head AIS scores of 6 were excluded. Patients with missing dmographics or vital signs were also excluded. LT-BI was defined as severe TBI that required burr hole evacuation or craniotomy within 24 h of hospital arrival [8]. Non-surviving patients with a head AIS score of 5 and an ISS lower than 34 who were considered dead due to isolated severe TBI were also defined as having LT-BI in order to reduce survivor bias.

### Assessment of consciousness levels in the prehospital setting

Patients with no eye opening (E1) on GCS at the first measurement in the prehospital setting were selected from the entire population for the purposes of predicting LT-BI. E1 on GCS equals three-digit code on Japan Coma Scale (JCS) signifying that they were not arousable even with forceful stimulation. Because the JCS in the prehospital setting must be scored and recorded on the JTDB sheet, we used JCS to evaluate consciousness levels in the prehospital setting [9]. Assessment of consciousness levels based on the JCS are useful predictors of outcome in stroke patients [10].

### Cushing’s sign

Although hypertension, bradycardia, and respiratory irregularity are the triad of Cushing’s sign [1], respiratory irregularity was not available due to the subjective data.

The first measurement data of systolic blood pressure (pSBP) and heart rate (pHR) in the prehospital setting were compared between the LT-BI and non LT-BI groups in order to examine the effect of hypertension and bradycardia, focusing on patients with disturbed level of consciousness.

### Statistical analysis

First, the LT-BI and non LT-BI group patients were compared. Categorical variables are shown as frequencies or percentages, whereas continuous variables are presented as mean and standard deviation values or median and interquartile range values depending on their distributions. Categorical variables were compared using chi-square analysis. Student’s t test was used to assess continuous variables with normal distributions accompanied by Cohen’s *d* effect size value, and the Mann–Whitney U test was used to evaluate variables with non-normal distributions.

The ability of accuracy and predictability of LT-BI was estimated based on area under the receiver operating characteristic curve (AUROC) and multiple logistic regression analysis focusing on pSBP, pHR, and the combination of pSBP and pHR. Multiple logistic regression analysis was performed employing the forced entry method. Odds ratio and 95% confidence interval (CI) for LT-BI in each category based on the pSBP and pHR were described after adjusting for potential confounders including age, gender, chest AIS score (≥4 vs. <4), abdomen AIS score (≥4 vs. <4), and extremity AIS score (≥4 vs. <4), considering patients with pSBP of 100 to 139 mmHg and pHR of 60 to 99 bpm as the reference group [4]. *P* values of <0.05 were considered statistically significant. All analyses were performed using IBM SPSS Statistics 22 (IBM SPSS, Chicago, IL, U.S.A.).

## Results

### Study participants and baseline characteristics

**Fig. 1:**
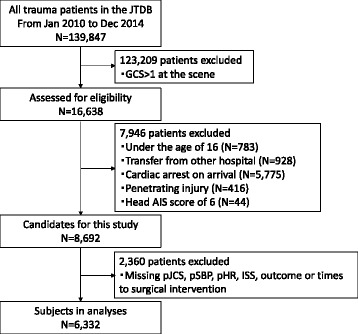
Flow diagram of patient inclusion/exclusion criteria

Of 139,847 patients registered in the JTDB during the 5-year study period, 6332 trauma patients met the inclusion criteria. Figure 1 shows the derivation of the final study population. Basic demographics of the LT-BI and the non LT-BI groups are summarized in Table 1. Of 6332 eligible patients, 1859 (29%) exhibited LT-BI. In terms of Cushing’s sign, the LT-BI group demonstrated higher pSBP (152 ± 40 mmHg vs. 129 ± 35 mmHg, *P* < 0.001, Cohen’s *d* effect size value of 0.63), and lower pHR (84 ± 23 bpm vs. 90 ± 24 mmHg, *P* < 0.001, Cohen’s *d* effect size value of 0.25).

**Table 1:** Baseline characteristics of the LT-BI and non LT-BI groups

	LT-BI *N* = 1859	Non LT-BI *N* = 4473	*P*-value
Age (year), mean ± SD	62 ± 20	54 ± 22	<0.001^*^
Female, n (%)	599 (32)	1,272 (28)	0.003^†^
pSBP (mmHg), mean ± SD	152 ± 40	129 ± 35	<0.001^*^
pHR (beats/min), mean ± SD	84 ± 23	90 ± 24	<0.001^*^
Chest AIS ≥ 4, n (%)	158 (9)	957 (21)	<0.001^†^
Abdomen AIS ≥ 4, n (%)	10 (0.5)	154 (3)	<0.001^†^
Extremity AIS ≥ 4, n (%)	36 (2)	292 (7)	<0.001^†^
ISS, median (IQR)	25 (25, 29)	22 (16, 34)	<0.001^‡^
Hospital mortality, n (%)	1,075 (58)	982 (22)	<0.001^†^

*LT-BI* life-threatening brain injuries requiring immediate neurosurgical intervention, *SD* standard deviation, *pSBP* systolic blood pressure in the prehospital setting, *pHR* heart rate in the prehospital setting, *AIS* Abbreviated Injury Scale, *ISS* Injury Severity Score, *IQR* interquartile range

^*^Student’s *t* test

^†^chi-square analysis

^‡^Mann–Whitney U test

### Effect of hypertension or bradycardia on prediction of LT-BI in the prehospital setting

**Fig. 2:**
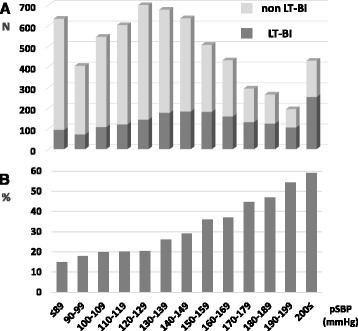
The number of patients and percentages presenting LT-BI based on pSBP. The number of patients with or without presenting LT-BI according to increments of 10 of pSBP (**a**). Percentages of those presenting with LT-BI (**b**). *LT-BI:* life-threatening brain injuries requiring immediate neurosurgical intervention, *pSBP*: systolic blood pressure in the prehospital setting

**Fig. 3:**
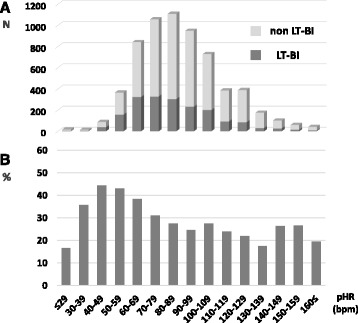
The number of patients and percentages presenting LT-BI based on pHR. The number of patients with or without presenting LT-BI according to increments of 10 of pHR (**a**). Percentages of those who were presenting with LT-BI (**b**). *LT-BI:* life-threatening brain injuries requiring immediate neurosurgical intervention, *pHR:* heart rate in the prehospital setting

The number of patients in increments of 10 of pSBP and pHR, and the percentages of those who presented with LT-BI are shown in Figs. 2 and 3, respectively. In terms of pSBP, the higher the pSBP, the higher the likelihood that the patients exhibited LT-BI (Fig. 2). The proportion of patients with LT-BI seemed to be higher in patients presenting with bradycardia (Fig. 3).

### AUROC values using pSBP, pHR, and the combination of pSBP and pHR for LT-BI in the prehospital setting

Although pSBP showed a higher predictability for LT-BI compared with pHR, both showed low accuracy, of which the AUROC was 0.666 (95% CI; 0.652-0.681, *P* < 0.001), and 0.578 (95% CI; 0.563-0.594, *P* < 0.001), respectively (Table 2). Then, the AUROC of the combination of pSBP and pHR was evaluated according to increments of 40 of pSBP and pHR. The AUROC of pSBP was highest among the 60 ≤ pHR ≤ 99 subgroup. However, it showed low accuracy, of which the AUROC was 0.680 (95% CI; 0.662-0.699, *P* < 0.001) (Table 2).

**Table 2:** AUROC values obtained using pSBP, pHR, and the combination of pSBP and pHR

	AUROC (95% CI)	*P*-value
pSBP overall	0.666 (0.652–0.681)	<0.001
pHR ≤ 59	0.651 (0.602–0.700)	<0.001
60 ≤ pHR ≤ 99	0.680 (0.662–0.699)	<0.001
100 ≤ pHR ≤ 139	0.643 (0.611–0.675)	<0.001
140 ≤ pHR	0.623 (0.532–0.715)	0.009
pHR overall	0.578 (0.563–0.594)	<0.001
pSBP ≤ 99	0.542 (0.491–0.592)	0.088
100 ≤ pSBP ≤ 139	0.577 (0.549–0.605)	<0.001
140 ≤ pSBP ≤ 179	0.573 (0.546–0.600)	<0.001
180 ≤ pSBP	0.611 (0.574–0.648)	<0.001

*AUROC* area under the receiver operating characteristics curve, *pSBP* systolic blood pressure in the prehospital setting, *pHR* heart rate in the prehospital setting, *CI* confidence interval

### Positive predictive value, sensitivity, and specificity of the combination of pSBP and pHR for LT-BI

**Fig. 4:**
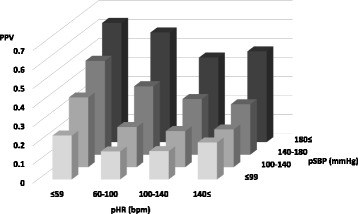
Positive predictive value of LT-BI. Positive predictive value of LT-BI according to the combination of pSBP and pHR. *LT-BI:* life-threatening brain injuries requiring immediate neurosurgical intervention, *pSBP:* systolic blood pressure in the prehospital setting, *pHR:* heart rate in the prehospital setting, *PPV:* positive predictive value

The proportion of those who presented with LT-BI according to the combination of increments of 40 of pSBP and pHR are shown in Fig. 4 accompanied by showing sensitivity and specificity (Table 3). The higher the pSBP and the lower the pHR, the more likely that the patients showed LT-BI.

**Table 3:** Sensitivity and specificity of LT-BI according to the combination of pSBP and pHR

		Sensitivity (95% CI)	Specificity (95% CI)
pSBP ≤ 99	pHR ≤ 59	0.011 (0.007–0.015)	0.985 (0.984–0.987)
	60 ≤ pHR ≤ 99	0.042 (0.035–0.052)	0.900 (0.896–0.903)
	100 ≤ pHR ≤ 139	0.030 (0.023–0.037)	0.931 (0.928–0.934)
	140 ≤ pHR	0.006 (0.004–0.010)	0.989 (0.988–0.991)
100 ≤ pSBP ≤ 139	pHR ≤ 59	0.034 (0.028–0.041)	0.975 (0.973–0.978)
	60 ≤ pHR ≤ 99	0.190 (0.175–0.206)	0.706 (0.700–0.713)
	100 ≤ pHR ≤ 139	0.065 (0.056–0.076)	0.885 (0.881–0.889)
	140 ≤ pHR	0.006 (0.003–0.010)	0.990 (0.989–0.992)
140 ≤ pSBP ≤ 179	pHR ≤ 59	0.042 (0.036–0.049)	0.982 (0.979–0.985)
	60 ≤ pHR ≤ 99	0.231 (0.215–0.246)	0.829 (0.823–0.836)
	100 ≤ pHR ≤ 139	0.072 (0.062–0.082)	0.928 (0.924–0.932)
	140 ≤ pHR	0.009 (0.006–0.013)	0.989 (0.988–0.991)
180 ≤ pSBP	pHR ≤ 59	0.023 (0.018–0.026)	0.994 (0.993–0.996)
	60 ≤ pHR ≤ 99	0.178 (0.166–0.189)	0.946 (0.941–0.951)
	100 ≤ pHR ≤ 139	0.055 (0.047–0.063)	0.972 (0.968–0.975)
	140 ≤ pHR	0.006 (0.004–0.008)	0.997 (0.996–0.998)

*LT-BI* life-threatening brain injuries requiring immediate neurosurgical intervention, *pSBP* systolic blood pressure in the prehospital setting, *pHR* heart rate in the prehospital setting

### Predictive models for LT-BI using pSBP, pHR, and the combination of pSBP and pHR

OR and 95% CI for LT-BI based on pSBP, pHR, and the combination of pSBP and pHR were described after adjusting for potential confounders including age, gender, chest AIS score (≥4 vs. <4), abdomen AIS score (≥4 vs. <4), and extremity AIS score (≥4 vs. <4) considering the patients with pSBP of 100 to 139 mmHg and pHR of 60 to 99 as the reference group.

Patients who were hypotensive in the prehospital setting were found to be less likely to have LT-BI compared with normotensive patients (Table 4A). Patients who were hypertensive or presenting with bradycardia in the prehospital setting were found to be more likely to have LT-BI compared with normotensive patients or patients with a normal range of heart rates (Table 4A, B).

**Table 4:** OR and 95% CI for LT-BI based on pSBP (A) and pHR (B)

Variables	Adjusted OR (95% CI)	*P*-value
A		
pSBP ≤ 99	0.78 (0.64–0.95)	0.013
100 ≤ pSBP ≤ 139	1.00 (reference)	
140 ≤ pSBP ≤ 179	1.68 (1.47–1.94)	<0.001
180 ≤ pSBP	3.21 (2.70–3.81)	<0.001
B		
pHR ≤ 59	1.67 (1.37–2.03)	<0.001
60 ≤ pHR ≤ 99	1.00 (reference)	
100 ≤ pHR ≤ 139	0.90 (0.78–1.03)	0.109
140 ≤ pHR	1.20 (0.85–.1.70)	0.292

Variables in the model were age, gender, chest AIS score (≥4 vs. <4), abdomen AIS score (≥4 vs. <4), and extremity AIS score (≥4 vs. <4), considering the patients with pSBP of 100 to 139 mmHg (A) and pHR of 60 to 99 mmHg (B) as the reference group, respectively

*OR* odds ratio, *CI* confidence interval, *LT-BI* life-threatening brain injuries requiring immediate neurosurgical intervention, *pSBP* systolic blood pressure in the prehospital setting, *pHR* heart rate in the prehospital setting, *AIS* Abbreviated Injury Scale

**Fig. 5:**
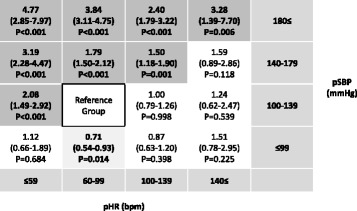
Adjusted OR of LT-BI. OR and 95% CI after adjusting for age, sex, chest AIS score (≥4 vs. <4), abdomen AIS score (≥4 vs. <4), and extremity AIS score (≥4 vs. <4), considering the patients with pSBP of 100 to 139 mmHg and pHR of 60 to 99 bpm as the reference group for LT-BI based on pSBP and pHR. *OR:* odds ratio, *LT-BI:* life-threatening brain injuries requiring immediate neurosurgical intervention, *CI:* confidence interval, *AIS:* Abbreviated Injury Scale, *pSBP:* systolic blood pressure in the prehospital setting, *pHR:* heart rate in the prehospital setting

OR and 95% CI for LT-BI focusing on the combination of pSBP and pHR is shown in Fig. 5. The higher the pSBP and the lower the pHR, the higher the predictability of LT-BI after adjusting for severity of injury to other body regions (Fig. 5).

## Discussion

Our study has found that the combination of hypertension and bradycardia, which are the components of Cushing’s sign, with GCS for eye opening of 1 in the prehospital setting was a weak but significant predictor of LT-BI, defined by the necessity for burr hole evacuation or craniotomy within 24 h of hospital arrival, or death due to possible isolated severe TBI.

Although pSBP and pHR showed low accuracy, pSBP was more predictive of LT-BI compared with pHR, which may be explained by the fact that the simultaneous onset of hypertension and tachycardia was a better indicator of impaired brain perfusion at an early phase during endoscopic neurosurgical procedures [11]. Hence, the combination of pSBP and pHR could be helpful to predict LT-BI among patients without eye opening, as Reisner et al. reported that patients with abnormal SBP, HR, and GCS in the prehospital setting were significantly more likely to have high mortality compared with the single parameter in TBI patients [12]. Our results showed that even subjects with the highest pSBP and lowest pHR had a positive predictive value of 0.61 and an adjusted odds ratio of only 4.77 versus the reference group. Also, an AUROC of around 0.6 corresponds to a fairly weak test. Cushing’s sign in the prehospital setting is a weak but significant predictor of the need for immediate neurosurgical intervention.

Prediction of the necessity for aggressive intervention against intracranial hypertension using simple vital signs in the prehospital setting can be helpful by leading to earlier therapeutic management, since appropriate early care is important to improve outcomes in TBI patients [13]. However, there have been no major investigations of Cushing’s sign predicting immediate neurosurgical intervention in TBI patients. Previously, many investigators reported the prognostic factors or models in TBI patients at an early stage after injury using vital signs, including GCS or pupillary reaction [4, 5, 12, 14, 15]. Among them, either prehospital hypertension or prehospital hypotension were revealed to be associated with higher mortality due to possible intracranial hypertension or paroxysmal sympathetic hyperactivity and impaired cerebral blood flow [3–5, 12]. To the best of our knowledge, our investigation is the first to describe Cushing’s sign in the prehospital setting predicting a life-threatening condition in TBI patients. Although early bifrontotemporoparietal decompressive craniectomy in diffuse TBI is associated with poor outcomes [16], early neurosurgical intervention in severe TBI would improve outcomes, especially in younger patients and those with higher GCS scores [17, 18]. Among isolated TBI patients who required neurosurgical intervention, the earlier the intervention, the better the survival rate [19]. Regardless of neurosurgical or medical intervention, since early appropriate care is inevitably associated with better outcomes, early prediction of LT-BI would enable us to determine earlier neurosurgical consultation and therapeutic intervention.

Considering the clinical situations, Cushing’s sign with disturbed level of consciousness in prehospital setting does not have high predictability enough to always warrant neurosurgical intervention, however, perhaps the findings could be used to support empiric medical treatment and intensive care of presumed increased intracranial pressure in a resource-poor environment in which invasive pressure monitoring or operation room were not readily available.

Our study have several limitations. First, since this was a nationwide retrospective study, many patients were excluded due to unavailability of vital signs in the prehospital setting. Second, our definition of LT-BI might not have been appropriate because the timing and cause of death in those with AIS scores of 5 and ISS lower than 34 were unknown. Third, because of the heterogeneity of TBI, computed tomography findings should have been taken into consideration in addition to head AIS score when evaluating severity of TBI.

Nevertheless, we described the impact of Cushing’s sign in the prehospital setting for predicting LT-BI, which can lead to earlier management and therapeutic intervention.

## Conclusions

In this large nationwide observational study, we demonstrated that prehospital Cushing’s sign with disturbed level of consciousness in trauma patients was a weak but significant predictor of the need for immediate neurosurgical intervention.

## Abbreviations

AIS: Abbreviated injury scale
AUROC: Area under the receiver operating characteristics curve
CI: Confidence interval
GCS: Glasgow coma scale
ISS: Injury severity score
JCS: Japan coma scale
JTDB: Japan Trauma Data Bank
LT-BI: Life-threatening brain injuries requiring immediate neurosurgical intervention
pHR: Heart rate in the prehospital setting
pSBP: Systolic blood pressure in the prehospital setting
TBI: Traumatic brain injury
